# Increasing the smoking cessation success rate by enhancing improvement of self-control through sleep-amplified memory consolidation: protocol of a randomized controlled, functional magnetic resonance study

**DOI:** 10.1186/s40359-025-02482-w

**Published:** 2025-02-22

**Authors:** Sarah Gerhardt, Michaela Kroth, Alexandra Seeger, Roland Schmitt, Heiner Fritz, Lorena Diring, Yury Shevchenko, Karen D Ersche, Gordon Feld, Sabine Vollstädt-Klein

**Affiliations:** 1 Department of Addictive Behaviour and Addiction Medicine, Medical Faculty Mannheim, Central Institute of Mental Health, University of Heidelberg, PO Box 12 21 20, D-68072 Mannheim, Germany; 2 Department of Clinical Psychology, Medical Faculty Mannheim, Central Institute of Mental Health, University of Heidelberg, Mannheim, Germany; 3 Research Methods, Assessment, and iScience, Department of Psychology, University of Konstanz, Konstanz, Germany; 4 Department of Psychiatry, University of Cambridge, Cambridge, UK; 5 Department of Systems Neuroscience, University Medical Center Hamburg-Eppendorf, Hamburg, Germany; 6 Mannheim Center for Translational Neurosciences (MCTN), Medical Faculty Mannheim, Heidelberg University, Mannheim, Germany; 7 German Center for Mental Health (DZPG), partner site Mannheim-Heidelberg-Ulm, Mannheim, Germany

**Keywords:** Addiction, Tobacco use disorder, Cognitive training, Habits, Cognitive control

## Abstract

**Background:**

Tobacco use disorder (TUD) remains a global health crisis characterized by high relapse rates despite extensive cessation efforts. This study aims to enhance treatment outcomes by addressing the cognitive and neural imbalances associated with habitual and goal-directed behaviours among individuals with TUD. We hypothesise that by integrating high-intensity interval training (HIIT), cognitive remediation treatment (CRT) via app-based chess training and a standard smoking cessation program (SCP) for cognitive control and sleep quality will be improved, thereby facilitating smoking cessation.

**Methods:**

The study will enrol 140 treatment-seeking smokers aged 18–65 years who meet the DSM-5 criteria for TUD. The participants will be randomly assigned to four groups: CRT + HIIT in the morning, CRT + HIIT in the evening, HIIT alone in the morning, and HIIT alone in the evening. Assessments will be conducted at baseline (T1), postintervention (T2), and at a three-month follow-up (T3) at the Central Institute of Mental Health in Mannheim, Germany. The primary outcomes include abstinence days or amount of alcohol consumed in cases of relapse, as well as craving reduction. Secondary outcomes include improvements in cognitive functions (working memory, response inhibition, and cognitive control), measured through neuropsychological tasks, functional magnetic resonance imaging (fMRI), polysomnography, and self-report questionnaires. The repeated-measures design allows for within-subject comparisons to evaluate intervention effectiveness.

**Discussion:**

This study aims to provide insights into the mechanisms through which combined CRT and evening HIIT, alongside improvements in sleep quality, can enhance smoking cessation outcomes. The hypothesised benefits on cognitive control and neural activity changes are expected to support better treatment adherence and reduced relapse rates among individuals with TUD. Addressing potential challenges such as high dropout rates through comprehensive participant support is crucial for the study’s success. Findings from this research could inform future therapeutic strategies for TUD, potentially advancing addiction treatment approaches. The integration of novel interventions with established cessation programs underscores the study’s significance in exploring holistic approaches to improving public health outcomes related to tobacco addiction.

**Trial registration:**

Registered at clinicaltrials.gov/ct2/show/NCT05726045 (Date 04.04.2024).

**Supplementary Information:**

The online version contains supplementary material available at 10.1186/s40359-025-02482-w.

## Background

Tobacco is the second most commonly used psychoactive substance, with over one billion smokers worldwide [1]. With frequent use, nicotine, the addictive compound in tobacco, can lead to tobacco use disorder (TUD) [2]. Despite the known comorbidities associated with regular tobacco use as well as the desire to quit, many smokers encounter difficulties in doing so. Consequently, it is not surprising that approximately 75% of smokers who voluntarily attempt to quit relapse after six months [3]. This phenomenon may be attributed to an imbalance between the flexible goal-directed system and the more rigid habit system in individuals with TUD, leading to a continuation of smoking [4, 5]. This perspective suggests that addictive behavior arises through a transition from initially goal-directed (purposeful) smoking to habitual, and eventually compulsive, use [6]. This is underpinned by neural processes, namely a dysfunction in fronto-striatal circuitry ( [7, 8]). As such, prefrontal cognitive control mechanisms (e.g., in the dorsolateral prefrontal cortex (DLPFC) and inferior frontal gyrus (IFG)) support goal-directed behavior, whereas habitual behavior is underpinned by more dorsal networks, including the putamen and the premotor cortex ( [9–11]). Research has demonstrated that patients with substance use disorder (SUD) have reduced cognitive function in multiple domains, including working memory, inhibition, problem-solving, cognitive flexibility, and judgment formation [12], with cognitive decline and impairments also observed in heavy smokers [13].

The cognitive remediation treatment (CRT) approach is believed to enhance cognitive abilities related to the domains of inhibition, decision-making, working memory, cognitive flexibility, and attention [14]. In the past, CRT has been successfully used to treat subjects with SUD and therefore could also reduce habitual behavior in subjects with TUD [15, 16]. There is also significant evidence suggesting that playing the game of chess enhances cognitive control and inhibitory capacity in patients with schizophrenia by strengthening executive functions [17, 18]). Regular chess training has also been shown to improve the cognitive impairments associated with SUD, including problem-solving skills, mental flexibility, and working memory [19], with this training also requiring the use of abilities such as problem-solving, environmental awareness, and the ability to react to unexpected changes. Previous functional magnetic resonance imaging (fMRI) studies have revealed that the DLPFC, a region relevant to addictive behavior [11], as well as several other brain regions, including premotor, parietal, temporal, and occipital cortices, are activated when players are actively solving chess-related situations ( [20–22]). Building on previous studies involving subjects with cocaine use disorder, it may be conceivable that chess-based cognitive remediation treatment (CB-CRT) has the potential to enhance inhibitory control and executive function, leading to decreased impulsivity ( [23, 24]). Consequently, this reduction in impulsivity may positively impact therapy outcome measures, for example, through reducing the likelihood of relapse. Two addiction mechanisms have been proposed to further contribute to the understanding of differential therapy outcomes.

First, psychoactive substances, especially nicotine, can impact circadian rhythms through behavioral entrainment, thereby altering an individual’s optimum performance [25]. This alteration can lead to degraded alertness and attention through affecting the prefrontal cortex [26]. Previous studies have shown that sleep deprivation can increase smoking behavior and that the prevalence of sleep disturbances is greater among smokers ( [27, 28]). Sleep can be categorized into five stages: wakefulness, rapid eye movement (REM) sleep, and nonrapid-eye-movement (NREM) sleep [29]. Quiet wakefulness is characterized by a predominance of alpha waves, whereas REM sleep is characterized by mixed frequency, muscle atonia, and rapid eye movement. NREM sleep is described by slower frequencies, k-complexes, and large negative waves followed by a slower positive wave; NREM sleep can be referred to as slow-wave sleep (SWS) [30]. Both SWS and REM sleep are independently and homeostatically regulated and depend on an individual’s internal ‘circadian clock’ [31]. Studies have demonstrated that sleeping after exposure therapy results in better therapy outcomes ( [32, 33]), suggesting that therapy outcomes could be improved by improving sleep quality [34]. Memory consolidation processes occur during sleep after a new memory is acquired, leading to a transformation from transient to long-term memory storage due to the repeated replay of the learned trace ( [35–37]). Given the significance of sleep in addictive behavior and corresponding therapeutic interventions, it can be concluded that improved sleep quality may positively affect substance abstinence. On these grounds, a successful smoking cessation program should include interventions to enhance sleep quality [38]. Rodent studies have shown that this repeated replay in memory consolidation originates from the hippocampus and then spreads to other parts of the brain. In humans, sleep-dependent memory consolidation is suggested to originate from targeted memory consolidation [39–41].

Second, previous research has demonstrated a greater risk of developing cardiovascular diseases in individuals with SUD, which is attributed to regular substance use, poor nutrition, and obesity. To address this concern, high-intensity interval training (HIIT) can be administered as a therapeutic intervention. HIIT is a relatively short interval training involving interleaved, short blocks of exercises at high or low intensity. Its goal is to reach at least 80% of one´s maximum heart rate. Interestingly, study results indicate not only improved aerobic power and a decreased risk of developing lifestyle diseases but also enhanced inhibitory control as a result of HIIT interventions [42]. In addition, greater physical fitness has been associated with improved quality of life and reduced craving levels [43]. Observations suggest that HIIT has a beneficial effect on sleep quality and efficiency [44], as it has been observed that physical exercise has the potential to improve sleep [45]. Physical activity in the early evening can also lead to more time spent in NREM sleep, more slow waves, and increased sleep spindles, resulting in an enhanced opportunity for memory consolidation. Recent studies have shown that exercise intensity positively modulates the effect of memory consolidation and results in improvements in impaired sleep-dependent memory consolidation in schizophrenic patients ( [46, 47]). This makes HIIT an ideal candidate to promote memory consolidation indirectly by improving sleep quality.

Overall, add-on interventions to standard smoking-cessation programs, such as CRT, show promise in increasing the likelihood of tobacco abstinence through promoting the improvement of self-control via sleep-amplified memory consolidation of the interventions themselves.

## Methods

### Aims and objectives

**Fig. 1:**
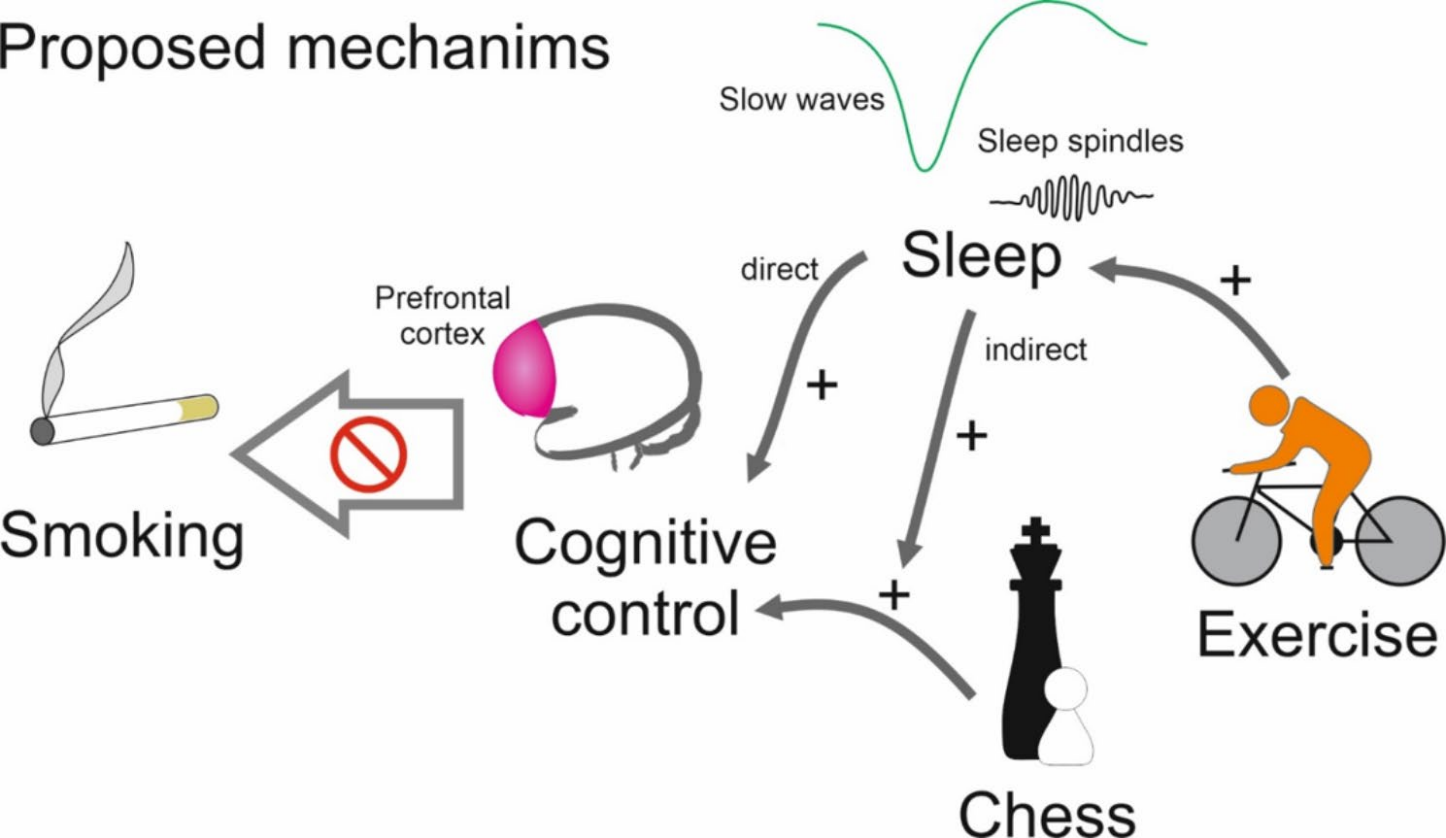
Schematic display of the study rational. The study aims to improve treatment outcomes in participants with TUD by enhancing cognitive control and sleep quality, thereby counteracting the hypothesised imbalance in regulatory control towards the habit system

This randomized controlled study aims to improve treatment outcomes in participants with TUD by enhancing cognitive control and sleep quality, thereby counteracting the hypothesised imbalance in regulatory control towards the habit system (see Fig. 1). This study further aims to identify the most effective pathway by which increased cognitive control promotes smoking cessation and thus reduces the risk of relapse. Two interventions will therefore be tested alongside the standard smoking cessation program, resulting in four intervention groups. One intervention will focus on improving sleep quality through app-based HIIT either in the morning or the early evening. The other intervention will involve CRT via an app-based chess training program either in the morning or early evening.

We hypothesise that a combination of CRT and HIIT in the evening will enhance treatment outcomes to a greater extent than sleep enhancement via HIIT alone or a combination of CRT and HIIT in the morning. This will be reflected in more abstinence days or fewer cigarettes smoked following relapse. Furthermore, we hypothesise that improved cognitive control is mirrored in improved behavioral results in neuropsychological tasks and changes in neural activity, e.g., during tasks assessing response inhibition or working memory. We predict that these changes are associated with both craving and treatment outcomes.

### Hypotheses

1. Primary hypotheses and treatment outcomes:

- A combination of CRT + sleep enhancement (i.e., CRT + HIIT early evening) as a six-week add-on therapy enhances treatment outcomes (i.e., relapse and craving) in smokers in comparison to CRT (i.e., CRT + HIIT morning) or sleep enhancement (i.e., no CRT + HIIT early evening) alone.
- The changes in neural measures of response inhibition, working memory and network connectivity in the salience network following the six-week treatment from baseline will be associated with self-reported craving and perceived stress, as well as treatment outcomes.

2. Secondary hypotheses and mechanisms:

Improvement in cognitive function in smokers.

c. CRT, as a six-week add-on training, improves cognitive function in smokers (i.e., CRT + HIIT in the morning versus HIIT in the morning).

d. Sleep enhancement (induced by HIIT in the evening) as a six-week add-on training improves cognitive function in smokers (i.e., HIIT in the evening versus HIIT in the morning).
e. The combined effect of sleep enhancement and CRT (i.e., the interaction effect of CRT × sleep enhancement) increases cognitive function even further.

Improvement in neuronal abnormalities.

f. CRT alleviates neural dysfunction during cognitive performance in smokers (i.e., CRT + HIIT in the morning versus HIIT in the morning).
g. Sleep enhancement as a six-week add-on therapy ameliorates neural dysfunction during cognitive performance in smokers (i.e., HIIT in the evening versus HIIT in the morning), as reflected by neural measures of response inhibition, working memory and connectivity strength within the salience network.
h. The combined effect of both training methods is even greater (i.e., the effect of CRT + HIIT in the evening versus HIIT in the morning).
i. CRT as well as sleep enhancement (i.e., HIIT in the early evening) as a six-week add-on therapy reduces connectivity strength in the salience network.

### Setting

Treatment-seeking participants with TUD according to the fifth version of the Diagnostic and Statistical Manual of Mental Disorders (DSM-5; [48]) will be randomised into four intervention arms. Assessments will take place before (baseline, T1) and after (second investigation day; T2) the six-week smoking cessation program, with a follow-up assessment (T3) three months later. A sport medical examination will be conducted at baseline at an outpatient cardiology clinic, while the other assessments will be conducted at the Central Institute of Mental Health, Mannheim, Germany.

### Study population and design

The study will be advertised by public advertisements and posters in the local community, newspaper outlets, websites and word of mouth. Interested candidates will undergo a short telephone screen to assess study eligibility and receive information about the study content and organization. A physical examination will then be conducted to ensure that participants are able to perform HIIT. Treatment-seeking smokers (men and women) aged between 18 and 65 years will be included in the study. The inclusion criteria will require prospective participants to meet at least four of the eleven TUD criteria according to the DSM-5, have a body mass index of between 18 and 35, demonstrate sufficient German proficiency to understand questions (both orally and in writing), communicate with investigators, and provide written consent. Additionally, participants must pass a physical examination to ensure that their participation in HIIT is safe. The exclusion criteria will apply to participants with severe somatic or neurological diseases and psychiatric comorbidities other than TUD according to the International Classification of Diseases (ICD-10) and DSM-5. A diagnosis of a sleep disorder (such as restless leg syndrome or insomnia) can also result in exclusion. Additionally, shift workers and night workers will be excluded. Participants will be excluded if they meet any of the following additional criteria: pregnancy and/or breastfeeding; high alcohol intake (screening scores with a score of 4 or higher for women or 5 (or higher) for men [49]); positive drug screening (e.g., amphetamine, benzodiazepine, cocaine, opioids); recent use of psychotropic medications (within the last 14 days, except stable adjusted SSRIs); medications affecting sleep, TUD, or physical activity (e.g., sleep medication, corticosteroids, stimulants); thyroid dysfunction or thyroid stimulation hormone levels outside the normal range; high blood pressure (resting heart rate > 100); or common MRI exclusion criteria (metal and claustrophobia).

**Fig. 2:**
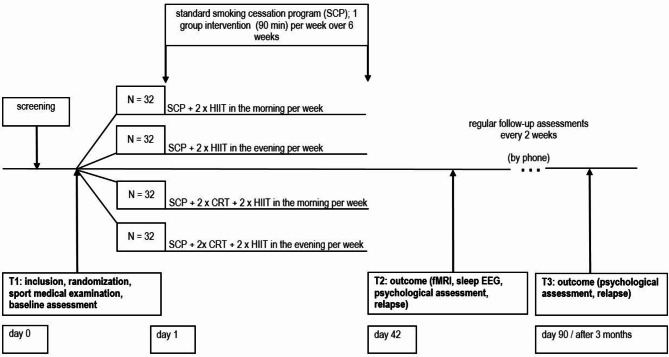
Study schedule and measurement time points. CRT: cognitive remediation treatment; fMRI: functional magnetic resonance imaging; HIIT: high-intensity interval training; SCP: standard smoking cessation program

A total of 128 participants will be randomized into four groups (4 × 32 smokers), receiving add-on interventions alongside a smoking cessation program (SCP) [50]. The randomization protocol will control for sex. Stratified (men/women) block randomization with permuted blocks of size 8 will be used. Groups 1 and 2 will receive CRT + HIIT either in the evening or morning, respectively. Group 3 and Group 4 will receive only HIIT in the evening or morning, respectively. Each subject will undergo three days of investigation. Assessments will occur before SCP (baseline, T1), after a six-week SCP (T2), and at a follow-up appointment three months later (T3). An in-house smartphone application will be used to remind participants about the exercises and to collect accompanying information, i.e., physical exertion (every training day), cigarette craving (3 times per week), and evaluation of the three interventions (three days per week). Instances of relapse and any sleep or physical problems can also be reported. These data will be stored on local servers at the institute, and no personal information will be collected. Between T2 and T3, biweekly telephone screenings will be conducted to assess abstinence or relapse, as well as the amount of cigarette consumption. See study schedule, Fig. 2.

### Sample size

The sample size was calculated via the software package G*Power (version 3.1.9.4 [51]) on the basis of the number of days of abstinence at posttreatment (day 42). With a sample size of 128 participants (32 participants per group), we achieve a power of 80%, assuming a minimum effect size of f = 0.25 in an ANOVA at a 5% alpha level. This ensures an optimal balance of sample size, information gain, and resource allocation [52]. To account for potential dropouts, three participants per group will be added, resulting in a total population of 140 participants.

### Interventions

Standard Smoking Cessation Program (SCP): The SCP will consist of group treatment sessions conducted once a week for one hour over a duration of six weeks for each subject. The program is grounded in behavioral therapy principles and will be facilitated by a qualified therapist [53].

Cognitive remediation treatment (CRT): The CRT will be administered as an app-based, chess-focused battery of tasks to be completed twice a week over a period of six weeks, with each session lasting 60 min. The CB-CRT was initially applied as an in-person group training and has since been adapted into a German version compatible with the GYMCHESS^®^ app (https://gymchess.com). This treatment approach utilizes a validated battery aimed at enhancing cognitive functions, including short-term memory, selective and focal attention, pattern recognition, visuospatial abilities, planning skills, and inhibition [54, 55]. In addition to the freely available GYMCHESS^®^ version, the participants will use a modified version with a fixed selection of exercises so that the same cognitive domains will be trained and that training for all participants will take place twice a week. No personal data will be stored during the use of the application. The date and time when the exercises were performed are recorded. Additionally, the number of completed exercises, (in-) correct answers, as well as the difficulty of the task are recorded.

**Fig. 3:**
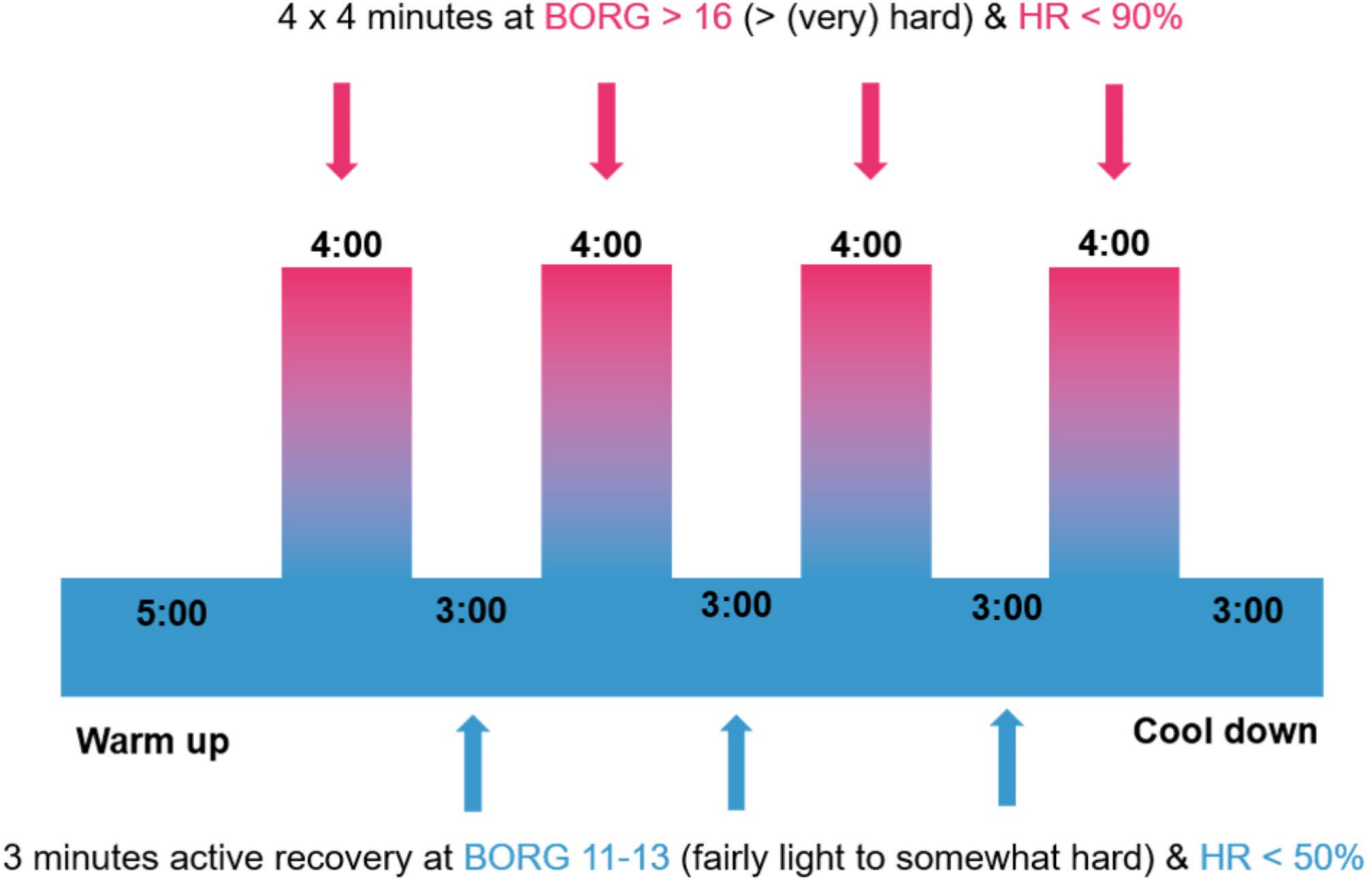
Training details of high-intensity interval training (HIIT). HR: Heart rate; Borg: Borg Scale to assess perceived exertion

High-intensity interval training (HIIT): This six-week, home-based program consists of four progressive stages, with each session beginning with a 5-minute warm-up and ending with a 3-minute cool-down. The workouts feature high-intensity intervals of 4 min, which are divided into four 1-minute segments of exercise and rest. Each high-intensity interval is followed by 3 min of active rest. Unlike traditional HIIT, this protocol emphasizes high-intensity functional movements that are adaptable to various fitness levels [56]. Low-intensity exercises (walking in place) are performed between high-intensity intervals. In week 1, the participants perform three intervals, each consisting of four sets of 15 s of exercise followed by 15 s of rest. After completing the four sets, there is a 3-minute active rest period. In week 2, a fourth interval is added; see Fig. 3. Weeks 3 and 4 maintain four intervals but modify the exercise/rest ratio to 20 s/10 seconds. Weeks 5 and 6 reduce the active rest to 2 min 30 s. The session durations are 26 min in week 1, 33 min in weeks 2–4, and 31.5 min in weeks 5–6. The participants are required to reach 90% of their maximum heart rate, which was determined during baseline physical exams. The Borg scale [57] is used to assess perceived exertion and ranges from a score between six (no exertion) and 20 (maximal exertion). HIIT sessions are conducted twice weekly, at least three hours before or after sleep, either in the morning or evening. Garmin^®^ (https://www.garmin.com/) fitness trackers and chest straps monitor heart rate, with data collected and visualized through Fitrockr Health Solutions (https://www.fitrockr.com/), a collaborative partner.

### Baseline Assessment (T1)

After recruitment, screening, and assessment of the inclusion and exclusion criteria, all participants are assigned to one of the four intervention groups and undergo a baseline assessment (T1). This includes obtaining fully informed written consent and conducting a diagnostic interview to validate TUD and any comorbid mental disorders [48]. Additionally, participants must undergo a physical examination to determine their suitability for participating in HIIT and to establish their individual maximum heart rate. The participants are then asked to complete various questionnaires, psychological assessments, and neurophysiological assessments, with an overview of the utilized questionnaires provided in Table 1.

Subsequently, participants undergo approximately one hour of fMRI, which includes two experiments (the N-back task and the stop-signal task (SST)), resting-state fMRI, and anatomical measurements. In addition, the participants undergo polysomnography.

SST: The SST is conducted during fMRI assessment to evaluate response inhibition [58]. The participants are instructed to respond to arrows, indicating left or right directions, by pressing the corresponding key on a remote control held in their hand with the respective thumb. In approximately 20% of the trials, a stop signal represented by an upwards-pointing arrow appears shortly after the presentation of a left or right arrow. The participants are required to withhold their response when the stop signal appears [59]. The task lasts for 13 min and comprises 320 go trials and 80 stop trials, with the aim of achieving an average of 50% stop successes and 50% stop errors. To maintain this balance, the time between the presentation of the go signal (left or right arrow) and the appearance of the stop signal (upwards-pointing arrow) is adjusted dynamically during the experiment. This adjustment, known as the stop signal delay time (SSD), is increased by 50 ms following a successfully inhibited response and decreased by 50 ms after failure. A longer SSD indicates better inhibitory performance. The reaction time and stop-signal reaction time are subsequently computed to estimate response latency in inhibition [60]. Test-retest reliabilities of neural activity during response inhibition were shown to be good [61].

N-back task: To assess working memory capacity, an N-back task with a block design will be conducted during fMRI examination [62]. The participants are required to respond to previously displayed numbers ranging from 1 to 4. In the 0-back condition, participants must press the corresponding key on the remote control for the currently displayed number. In the 2-back condition, participants are instructed to press the button corresponding to the stimulus (numbers 1–4) that appeared two stimuli before the current one. The retest reliability of this task has been shown to be very good [intraclass correlation coefficient of 0.90; 63], and the lack of learning effects during training sessions has been shown to not interfere with exercise outcomes [64].

Resting state: During the final fMRI examination, participants are instructed to fixate their gaze on a cross for an 8-minute resting-state measurement. Research has shown that resting-state measurements can be utilized to predict drug-related responses [65].

fMRI parameters: Scanning will be performed with a 3 T whole-body tomograph (MAGNETOM Prisma; Siemens, Erlangen, Germany). T2*-weighted multiband echo-planar images using a multiband acceleration factor of 6 are acquired in a transverse orientation 20° clockwise to the AC-PC line covering the whole brain with the following parameters: TR = 869 ms, TE = 38 ms, 60 slices, slice thickness = 2.4 mm, voxel size 2.4 × 2.4 × 2.4 mm^3, no interslice gap, field of view = 210 mm, matrix size 88 × 88, acquisition orientation T > C, interleaved slice order, acceleration factor slice = 6, flip angle = 58°, bandwidth = 1832 Hz/Px, prescan normalization, weak raw data filter, LeakBlock kernel, fat sat). The scanner sequences are provided by the Center for Magnetic Resonance Research, University of Minnesota, Minneapolis, MN, USA (https://www.cmrr.umn.edu/multiband/). In addition, a T1-weighted 3D Magnetization Prepared - RApid Gradient Echo dataset consisting of 208 sagittal slices (slice thickness 1 mm, 1 × 1 × 1 mm^3 voxel size, field of view 256 × 256 mm^2, TR = 2000 ms, TE = 2.01 ms, TI = 800 ms, flip angle = 8°) will be acquired. To examine brain iron content, a multiecho GRE imaging sequence will further be acquired (field of view = 220 mm, voxel size 0.9 × 0.9 × 1.4 mm^3, TR = 61 ms, 8 evenly spaced echoes (TE1/TE spacing = 4.5/5.5 ms), bandwidth = 560 (260) Hz/pixel, flip angle = 15°, acquisition time 7:51 min).

Polysomnography (PSG): A CE-certified system (Brain Vision ActiChamp-Plus) will be used to monitor sleep mechanisms. The setup includes 64 scalp electrodes, seven additional electrodes and a breathing belt designed for measuring sleep architecture and characterizing sleep oscillations. The additional electrodes monitor vertical and horizontal eye movements via electrooculography, muscle activity via electromyography, and heart activity via electrocardiography to provide a comprehensive understanding of sleep patterns and to identify/understand abstinence mechanisms during smoking cessation.

Neuropsychological assessments: Multiple neuropsychological assessments will be conducted. The Ravens Progressive Matrices Test [66] is a nonverbal test designed to assess logical thinking through a multiple-choice format. The participants are presented with patterns arranged in matrices and are required to identify the missing piece. The delay discounting task [67] is utilized to measure impulsive decision-making. The Participants make choices between smaller immediate rewards and larger delayed rewards. The Dimensional Card Sorting Task [68] assesses participants’ attentional bias and executive functions by requiring them to match cards to a target card on the basis of a changing rule. The Dot Probe Task [69] evaluates intrinsic attentional selection. Similar to the Dot Probe Task, the Stroop Task [70] measures selective attentional processing by asking participants to name the ink color of words that are incongruent with their meaning. For this study, a three-color Stroop task was implemented (yellow, red, blue), with congruent and incongruent stimuli, letters written in color, and smoking-associated or neutral words written in color. The continuous performance task [71] measures attentional vigilance by requiring participants to respond to specific target stimuli while inhibiting responses to distractors. Finally, the cue reactivity task [72] assesses cue reactivity by presenting participants with blocks of pictures containing either neutral or smoking-related stimuli and measuring craving and smoking desire after each block.

Questionnaires: Please see Table 1 for the questionnaires used throughout the study.

### Second investigation day (T2)

The second investigation day (T2) is conducted after the completion of the 6-week SCP. Similar to T1, participants are asked to complete different fMRI assessments, as well as neuropsychological assessments and questionnaires. A second PSG will also take place. A complete list of all the measurements is provided in Table 1.

### Follow-up (T3)

Three months after the second investigation (T2), the participants will receive the same self-rating questionnaires as for T1 and T2, see Table 1. Abstinence or relapse as well as nicotine consumption will be assessed during a final telephone interview. In addition, between T2 and T3, biweekly telephone interviews will take place to assess abstinence or relapse during the follow-up period of 90 days.

**Table 1:** Measurements and interventions

		T1	T2	I1-6	FU1-5	T3
**Baseline information and medical screening**	Sociodemographic data	x	(x)^*^			(x)^*^
	Internistic, neurological examination	x	(x)^*^			(x)^*^
	Drug/pregnancy urine screening	x	x			
	Menstrual cycle	x	x			
	Body Mass Index	x	x			x
	Carbon monoxide	x	x	x		
	Structured Clinical Interview for DSM-5	x				
	Sport medical examination	x				
	Edinburgh Handedness Inventory (73)	x				
**Questionnaires Smoking**	Questionnaire of Smoking Urges (74)	x	x			x
	Fagerstrøm Test of Cigarette Dependence (75)	x	x			x
	Craving Automated Scale for Cigarette (CAS-CS;	x	x			x
	adapted from CAS-A,(76))					
	Smoking Consequences Questionnaire (SCQ,(77))	x	x			x
	Wisconsin Smoking Withdrawal Scale (WSWS,(78))	x	x			x
	Cigarette Craving (Visual Analogue Scale)			x		
**Questionnaires Clinic**	Center for Epidemiological Studies Depression Scale (CES-D/ADS,(79))	x	x			x
	Perceived Stress Scale (PSS,(80))	x	x			x
	State-Trait-Anxiety Inventory (STAI,(81))	x	x			x
	PANAS Trait/State (82)	x	x			
	Barratt Impulsiveness Scale (BIS,(83))	x	x			x
	Creature of Habit Scale (COHS,(84))	x	x			x
	ASRS-V1.1 Screener (WHO, 2004)	x				
**Questionnaires Sleep**	Caffeine consumption questionnaire - revised	x	x			x
	(CCQ, (85))					
	Pittsburgh Sleep Quality Index (PSQI,(86))	x	x			x
	Global physical activity questionnaire (GPAQ, WHO)	x	x			x
	Munich Chronotype Questionnaire (MCTQ,(87))	x	x			x
	Stanford Sleepiness Scale (SSS,(88))	x	x			x
	Saint Mary's Hospital Sleep Questionnaire (89)	x	x			x
	Sleep Problems			x		
**Questionnaires Therapy expectation**	Goal Attainment scaling (90)	x	x			x
**Outcome consumption**	Form 90 interview (91)	x	x		x	x
	Time to relapse	x	x		x	x
	Days of abstinence	x	x	x	x	x
	Number of cigarettes	x	x	x	x	x
**Neuropsychological Assessment**	Delay Reward Discounting Task (67)	x	x			
	Dimensional Card Sorting Task (68)	x	x			
	Dot-probe (69)	x	x			
	Raven’s Progressive Matrices Test (66)	x				
	Cue reactivity (modified after (92)	x	x			
	(Nicotine) Stroop Task (modified after (70))	x	x			
	Continuous Performance Task (93)	x	x			
	Psychomotor Vigilance Task (94)	x	x			
**Functional magnetic resonance imaging**	Structural MRT	x	x			
	Resting state	x	x			
	mGRE	x	x			
	N-Back Task (62)	x	x			
	Stop-Signal Task (60)	x	x			
**Polysomnography**	Electroencephalography	x	x			
**Interventions**	Standard SCP plus CRT + HIIT			X		
	Standard SCP plus HIIT			x		
	Motivation and difficulty (HIIT, SCP) Visual Analogue Scale			x^**^		
	Borg Scale (57)			x^***^		
	Evaluation of each intervention (HIIT, CRT, SCP)					
	Visual Analogue Scale					

Note: T1 = first investigation day, T2 = second investigation day, I = Intervention (weeks 1 to 6), FU = biweekly follow-up period, T3 = final investigation day after three months, mGRE = multiecho GRE sequence, SCP = smoking cessation program, HIIT = high-intensity interval training. *updated if necessary. ** Motivation and difficulty of the intervention will be assessed after each intervention time point. ***Evaluation of all three interventions will be assessed weekly (‘does the intervention seem logical to you’, ‘how promising does the intervention appear to you’, ‘how likely would you recommend the intervention to a friend’, ‘how interested are you in participating in the training’, ‘how successful do you think the training will be’)

### Statistical analyses

Primary outcome measures include [1] treatment outcome (i.e., abstinence rates) after a six-week intervention phase compared with baseline; [2] improvement in cognition and thus changes in neural measures of response inhibition, working memory and salience network connectivity in relation to craving, perceived stress and treatment success. After preprocessing, including movement correction and normalization to the Montreal Neurological Institute template, the echo planar images will undergo spatial smoothing with a Gaussian kernel of 8 mm full width at half maximum. The analysis will be conducted at both the single-subject and group levels via the general linear model approach. For resting-state analysis, the seed region is the right anterior insula for the salience network, which is one of the two core regions anchoring the salience network [95]. At the second level of analysis, paired t tests will be used to observe the effects of groups and time (preintervention vs. postintervention within one group). Additionally, full factorial models will be employed. Regression models incorporating various clinical variables, such as the severity of TUD, will also be calculated. Correction for statistical testing will be performed via familywise error correction at the level of *P* < 0.05. EEG data during sleep will be analysed via an in-house script in python, resulting in hypnograms and information, such as the timing and length of sleep stages, as well as spectrograms, including information on activation and frequency at specific electrodes.

SPSS (Statistics for Windows, IBM Corp., Armonk, NY) and R (R: A language and environment for statistical computing. R Foundation for Statistical Computing, Vienna, Austria) will be utilized to analyse physiological (e.g., heat rate during HIIT), psychometric (e.g., questionnaire data) and neuropsychological data. Multivariate analyses of variances with repeated measures will be conducted to analyse the dependent variables. Additionally, linear regression models will be employed to examine the influence of confounding variables on changes in the dependent variables. Finally, Cox regression analyses, such as examining brain activation in the dorsolateral prefrontal and/or inferior frontal regions during inhibition, will be performed to evaluate associations with possible relapse.

## Discussion

Owing to the challenges faced by patients with TUD in maintaining abstinence, smoking remains a global health crisis and a leading cause of premature death [96]. This study aims to establish a therapeutic approach for improved treatment outcomes, including increased abstinence. Additionally, we aim to address this research gap by evaluating different intervention approaches to understand the imbalance between habitual and goal-directed behavior. Emerging evidence indicates an imbalance in habitual and goal-directed behavior among treatment-seeking smokers at both the neural and behavioral levels, suggesting that this imbalance can predict future relapses [65]. To achieve better treatment outcomes and assist treatment-seeking smokers in reaching their goals, we will implement two interventions in addition to the standard SCP. A notable aspect of our study is the implementation of outpatient HIIT and CRT, which has not been conducted in patients with TUD in previous studies. The outpatient setting allows participants to engage in the interventions individually within their familiar environment. As a contribution to the field, we aim to identify the mechanisms through which sleep enhancement and CRT may improve cognitive control, addressing a research gap regarding the neural mechanisms underlying the influence of cognitive control on abstinence. We hypothesise that the additional interventions will have differential beneficial effects on relapse rates and nicotine consumption by enhancing cognitive function. By utilizing our validated cognitive battery in the CRT intervention, we aim to enhance cognitive functioning in domains such as inhibition, working memory, attention, and decision-making. We anticipate further improvements in cognitive functions through the enhancement of sleep quality provided by early evening HIIT sessions. The use of a repeated-measures design can be considered a methodological strength, as it reduces error variance by allowing each patient to serve as their own control. This design will help to accurately assess the effectiveness of the interventions [97].

The potential limitation of this study could be the high dropout rate, as it involves a variety of tasks and interventions. To mitigate this risk, participants will be adequately supported throughout the conduct of the story and motivated to complete the study. Additionally, the numerous tasks involved may present challenges in integrating the findings, although they also provide an opportunity to explore the complexity of TUD.

## Electronic supplementary material

Below is the link to the electronic supplementary material.

Supplementary Material 1

## Data Availability

As the clinical and neuroimaging data are sensitive, data will not be made publicly available. Upon direct request by other researchers and in mutual agreements regarding data protection, anonymized data could be made available (e.g., MRI data after defacing). In the context of scientific research analysis, procedures and codes will be shared with other researchers upon request.

## Abbreviations

CRT: Cognitive remediation treatment
DSM: Diagnostic and Statistical Manual of Mental Disorders
DLPFC: Dorsolateral prefrontal cortex
fMRI: Functional magnetic resonance imaging
HIIT: High-intensity interval training
IFG: Inferior frontal gyrus
ICD: International Classification of Diseases
NREM: Nonrapid eye movement
REM: Rapid eye movement
SSRIs: Selective serotonin reuptake inhibitors
SWS: Slow-wave sleep
SCP: Smoking cessation program
SST: Stop-Signal Task
SUD: Substance use disorder
TUD: Tobacco use disorder
