# Supplementary material for: Increasing the smoking cessation success rate by enhancing improvement of self-control through sleep-amplified memory consolidation: protocol of a randomized controlled, functional magnetic resonance study
Source: BMC Psychol. 2025 Feb 22;13:157. doi: 10.1186/s40359-025-02482-w (PMC11847401; doi:10.1186/s40359-025-02482-w)
Supplement: Supplementary file 1 — Supplementary Material 1 [file 40359_2025_2482_MOESM1_ESM.doc]

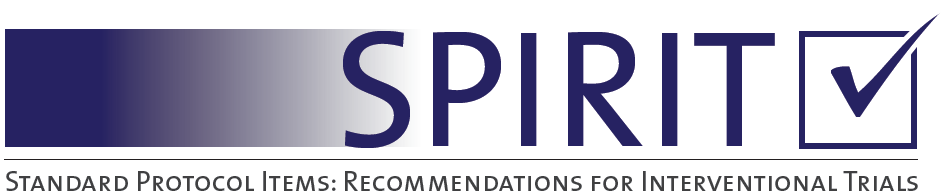


SPIRIT 2013 Checklist*

| Section/item | ItemNo | Description |
| --- | --- | --- |
| **Administrative information** | | |
| Title | 1 | Increasing the smoking cessation success rate by enhancing improvement of self-control through sleep-amplified memory consolidation: protocol of a randomized controlled, functional magnetic resonance study |
| Trial registration | 2a | Registered at clinicaltrials.gov/ct2/show/NCT05726045 |
|  | 2b | ./. |
| Protocol version | 3 | ./. |
| Funding | 4 | Funded by a grant from the Deutsche Forschungsgemeinschaft (DFG, German Research Foundation), TRR 265 Project-ID 402170461 (Heinz et al., Addict Biol. 2019). |
| Roles and responsibilities | 5a | KDE^1,4,5^, GF^2^, and SVK^1,6,7^ were responsible for the study design and the procurement of the study funding. SG^1^ drafted the manuscript with input from LD^2^, MK^2^, HF^1^, YS^1,3^, KDE^1,4,5^, GF^2^, and SVK^1,6,7^. All authors made substantial contributions to the manuscript. All authors read and approved the final manuscript.  ^1^ Department of Addictive Behaviour and Addiction Medicine, Central Institute of Mental Health, Medical Faculty Mannheim, University of Heidelberg, Mannheim, Germany  ^2^ Department of Clinical Psychology, Central Institute of Mental Health, Medical Faculty Mannheim, University of Heidelberg, Mannheim, Germany  ^3^ Research Methods, Assessment, and iScience, Department of Psychology, University of Konstanz, Germany.  ^4^ Department of Psychiatry, University of Cambridge, Cambridge, United Kingdom  ^5^Department of Systems Neuroscience, University Medical Center Hamburg-Eppendorf, Hamburg, Germany  ^6^ Mannheim Center for Translational Neurosciences (MCTN), Medical Faculty Mannheim, Heidelberg University, Mannheim, Germany  ^7^ German Center for Mental Health (DZPG), partner site Mannheim-Heidelberg-Ulm, Germany |
|  | 5b | Central Institute of Mental Health, J5, 68159 Mannheim, Germany |
|  | 5c | The funders and sponsor had no role in the study design; they will also not have a role in upcoming data collection, data analysis, data interpretation, or the writing of manuscripts. |
|  | 5d | Principal Investigators:  Sabine Vollstädt-Klein, Prof. Dr. (Lead Investigator), Gordon Feld, PD Dr., Karen D. Ersche, Prof. Dr. |
| Introduction |  |  |
| Background and rationale | 6a | See chapter ‘Background’ of the study protocol |
|  | 6b | See chapter ‘Background’ of the study protocol |
| Objectives | 7 | See chapter ’Methods` / ‘Aims and Objectives’ and ‘Hypotheses’ |
| Trial design | 8 | Randomized, controlled trial |
| Methods: Participants, interventions, and outcomes | | |
| Study setting | 9 | Germany, urban, one study site. |
| Eligibility criteria | 10 | See chapter ‘Methods’ / ‘Study population and design’ of the study protocol |
| Interventions | 11a | See chapter ‘Methods’ / ‘Interventions’ of the study protocol |
|  | 11b | Discontinuation in case the participant withdraws consent, does not wish to participate any more, or is not reachable via phone, mail during follow-up period. |
|  | 11c | Standardized and automated reminders for interventions / questionnaires via study application on smartphone. Bi-weekly, standardized telephone appointments until 3-month follow-up examination |
|  | 11d | ./. |
| Outcomes | 12 | See chapter ‘Methods’ ‘Hypotheses' |
| Participant timeline | 13 | See chapter ‘Methods’ / ‘Study population and design’ of the study protocol, figure 2, and table 1 |
| Sample size | 14 | See chapter ‘Methods’ / ‘Sample size’ |
| Recruitment | 15 | See chapter ‘Methods’ / Study population and design’ |
| **Methods: Assignment of interventions (for controlled trials)** | | |
| Allocation: |  |  |
| Sequence generation | 16a | A stratified (gender, binary) block randomization with permuted blocks of size 8 was used. |
| Allocation concealment mechanism | 16b | ./. |
| Implementation | 16c | generate the allocation sequence: Biostatistics Department, enrol participants: Addiction Department, will assign participants to interventions: Addiction Department |
| Blinding (masking) | 17a | no blinding. Participants are blinded with regards to expected outcomes following different interventions/ their respective group allocation |
|  | 17b | ./. |
| **Methods: Data collection, management, and analysis** | | |
| Data collection methods | 18a | Data will be collected and stored electronically (participant information, questionnaires, neuropsychological assessments: online using REDCap or an in-house study application, fMRI data: offline). Data collection will be done by trained and supervised staff members. |
|  | 18b | Participants will receive both, monetary compensation of their time, as well as individual results from sleep-EEG and anatomical MRI (non-medical focussed feedback) and their sports medical examination. In case of drop-outs, individual reasons will be assessed and documented if possible. |
| Data management | 19 | Data will be stored electronically (online for questionnaires and neuropsychological tasks using REDCap or an in-house study application). Data entry and storage includes mandatory data entry (‘required fields’) and prompts about process status (e.g. next steps) in order to standardize data collection and communication with participants as well as to minimize missing data or errors. Online data will be downloaded monthly. Data will be stored locally and backed-up hourly. Data collection and management will follow the general data protection regulation for Germany. |
| Statistical methods | 20a | See chapter ‘Methods’ / ‘Statistical analyses’ |
|  | 20b | Secondary analyses might include, for example, merging this data set with prior studies examining smokers. |
|  | 20c | Intention to treat (Cox) and, in addition, per protocol |
| **Methods: Monitoring** | | |
| Data monitoring | 21a | No DMC. |
|  | 21b | Interim-Analyses: e.g. for conference presentations. Premature termination of data collection: in case of expired funding before completion of data collection |
| Harms | 22 | Collecting: Asking about adverse events, also during follow-ups. Reporting: via clinical trials upon project termination or in publications. Managing: by providing professional help (e.g., psychotherapist, physician), by terminating study participation, . |
| Auditing | 23 | No periodic independent review |
| Ethics and dissemination | | |
| Research ethics approval | 24 | This study has been approved by the Ethics Committee II of the Medical Faculty Mannheim at the University of Heidelberg, Germany (Ethics approval number: 2018-625N) and is in accordance with the requirements of the World Medical Association’s Declaration of Helsinki. Amendments to study design, study personnel or others will be reviewed as well by the ethics board. |
| Protocol amendments | 25 | Major amendments (e.g. tasks, in-/exclusion criteria, sample size) to the protocol will be documented in clinical trials. |
| Consent or assent | 26a | Trained personnel, psychologists, and clinical staff using written informed consent documents that have been evaluated and approved by the ethics committee. These documents will be discussed with potential participants, and all questions will be answered. Subsequently, written informed consent will be obtained. |
|  | 26b | ./. |
| Confidentiality | 27 | Personal information will be stored separate from study data via DFN-AAI according to local data security regulations with limited access. Only study ID (pseudonymised) will be used in combination with assessed study data. All informed consent documents (paper documents) will be stored securely at the study site in locked file cabinets in areas with limited access. |
| Declaration of interests | 28 | none |
| Access to data | 29 | Scientific staff from department of principal investigators upon reasonable request. Collaboration partners upon reasonable request in and in accordance with participant consent. |
| Ancillary and post-trial care | 30 | Providing information regarding maintenance of abstinence, relapse prevention or further support. |
| Dissemination policy | 31a | Publications in peer-reviewed open-access journals, conference talks and presentations, in-house meetings. |
|  | 31b | Substantial contributors to the project / analyses / reporting will be included as authors. |
|  | 31c | Via open-access publications (results, study protocol). Access to data (anonymized) can be requested. |
| Appendices |  |  |
| Informed consent materials | 32 | Only available in German. Evaluated by the local Ethics Committee (incl. amendments) |
| Biological specimens | 33 | ./. |

* The SPIRIT checklist is copyrighted by the SPIRIT Group under the Creative Commons “[Attribution-NonCommercial-NoDerivs 3.0 Unported](http://www.creativecommons.org/licenses/by-nc-nd/3.0/)” license.
